# Quantifying service sector waste: insights from the Spanish economy

**DOI:** 10.1007/s11356-025-36758-w

**Published:** 2025-07-21

**Authors:** Mercedes Rodríguez, José A. Camacho

**Affiliations:** 1 Department of International and Spanish Economics, University of Granada, 18071 Granada, Spain; 2 Institute of Regional Development, University of Granada, 18071 Granada, Spain

**Keywords:** Waste generation, Input–output analysis, Indirect waste, Service sector, Circular economy, Spain

## Abstract

Global waste generation is projected to rise significantly in the coming decades, with high-income countries contributing disproportionately to per capita waste production. While the European Union’s Circular Economy Action Plans prioritize resource-intensive sectors, the service sector’s contributions to waste generation remain underexplored. This study offers a detailed assessment of waste generation in Spain, distinguishing between direct and indirect contributions as well as their domestic and foreign origins, with a particular focus on disaggregated data for service activities. By employing environmentally extended input–output modeling, this study quantifies not only the direct waste produced by service industries but also the substantial indirect waste embedded in their supply chains, thereby revealing the full extent of the sector’s contribution to national waste generation. While service industries generate relatively low levels of direct waste, the results reveal a substantial indirect footprint driven by upstream supply chains. These findings underscore the need to integrate disaggregated service sector data into waste accounting systems and to align circular economy policies with the sector’s full environmental impact. Building on these findings, this study contributes valuable knowledge of the service sector’s role in waste generation and highlights key areas for future research aimed at fostering sustainable economic practices.

## Introduction

Global waste generation is projected to reach 3.40 billion metric tons annually by 2050, with high-income countries consistently leading in per capita waste levels despite slower growth rates (Kaza et al. 2018). Within the European Union (EU), households and economic activities generated an average of 4991 kg of waste per capita in 2022, despite a modest 0.7% decline in total waste generation between 2004 and 2022 (Eurostat 2024). Spain’s per capita waste generation is broadly aligned with the EU average and shares structural similarities with other high-income, service-based economies. For instance, in 2022, Spain generated 1474 kg of waste per capita excluding major mineral wastes, a figure comparable to France’s 1451 kg per capita (Eurostat 2024). These similarities make Spain a relevant case study with potential implications beyond the national context.

Although the evolution of Spain’s total waste generation mirrors EU trends, the country’s waste management performance shows notable shortcomings. In 2022, the preparation for reuse and recycling rate of municipal waste stood at 39%, significantly below the EU average of 49%, and concerns have been raised about the underreporting of packaging waste, which could result in overestimated recycling rates. Furthermore, Spain remains approximately 40 percentage points above the 2035 EU landfill target (EEA 2025). These challenges underscore the need for more robust and sector-specific waste management strategies, underpinned by comprehensive analytical tools.

To address these challenges, the EU and Spain have pursued ambitious Circular Economy Action Plans, introduced in 2015 and expanded in 2020, which focus on resource-intensive sectors such as electronics, packaging, and construction materials (European Commission 2020; MITECO 2020). However, the service sector—encompassing activities such as education, healthcare, retail, transport, and hospitality—continues to be largely overlooked in circular economy policies. This omission reflects the persistent assumption that services are materially light and pose minimal environmental burdens. In Spain’s case, despite the structural importance of services, only one out of six priority sectors identified in its Circular Economy Strategy—tourism—is a service-related activity (MITECO 2020).

The EU’s waste prevention policies, encapsulated in the Waste Framework Directive (European Union 2008) and strengthened by Directive 2018/851 (European Union 2018), emphasize reducing waste quantities and mitigating adverse environmental and health impacts through reuse and extended product lifespans. Nevertheless, as noted by the European Environment Agency (EEA 2023), current indicators lack the granularity required for detailed sectoral analysis, particularly for services. Waste accounting frameworks remain focused on manufacturing, construction, and agriculture, partly due to historical data limitations. Consequently, a large share of the waste footprint remains invisible in sustainability assessments, hampering the development of inclusive and effective waste strategies.

The environmental impact of service industries has traditionally been underestimated, largely due to their low levels of direct waste generation compared to sectors such as manufacturing or construction. However, this narrow perspective overlooks the substantial waste generated indirectly through the complex and material-intensive supply chains that support service provision. Accurately accounting for these upstream flows is essential for the development of effective waste prevention and circular economy policies. A growing body of empirical research supports this view. In Japan, service industries have been identified as major contributors to incinerated and landfilled waste when their full supply-chain footprint is considered (Nakamura 2020). In Australia, Reynolds et al. (2016) and Fry et al. (2016) highlight significant amounts of commercial and industrial waste linked to service subsectors such as education, hospitality, and healthcare. In the UK, Salemdeeb et al. (2016) show that final demand for services indirectly drives large quantities of construction and demolition waste. At a global level, Tisserant et al. (2017) demonstrate that high-income, service-based economies exhibit disproportionately large waste footprints, driven largely by indirect flows embedded in international supply chains. These findings are corroborated in the USA by Meyer et al. (2020), who find that public administration and commercial services become prominent contributors to waste when shifting from direct generation to a consumption-based accounting perspective. Finally, regional evidence from Belgium confirms that services such as accommodation and food account for a substantial share of the waste footprint once indirect and stock-depletion components are included (Towa et al. 2021). These consistent results across diverse contexts provide a robust empirical foundation for hypothesizing that the service sector’s indirect waste generation is both significant and policy relevant.

The modeling of waste generation within economies has traditionally relied on input–output analysis, which captures intersectoral dependencies in national and global production systems. Over time, this approach has been adapted to assess environmental pressures by incorporating waste and resource use into economic accounts. Leontief’s pioneering work (Leontief 1970) introduced environmental extensions into input–output models, enabling the quantification of interindustry flows in monetary units while tracking environmental burdens in physical terms. Later developments disaggregated waste flows by type and treatment method (Nakamura 1999; Nakamura and Kondo 2002), while others integrated material flow accounting to improve data granularity (Nakamura et al. 2007). Hybrid approaches have also emerged, combining input–output analysis with life cycle assessment to evaluate environmental impacts across supply chains using both physical and economic data (Hendrickson et al. 2006; Suh and Huppes 2005). Despite these advances, service activities are often omitted due to the challenges of capturing their non-material outputs and the historical lack of disaggregated data (Liang et al. 2012). In this context, environmentally extended input–output (EEIO) models provide a particularly suitable framework for tracing both direct and upstream waste impacts. By linking final demand in service sectors to waste generated throughout supply chains, these models allow for a more accurate assessment of the environmental footprint associated with service-based economies.

From a theoretical standpoint, this study draws on EEIO modeling, rooted in Leontief’s intersectoral theory, and widely applied in environmental footprinting (Miller and Blair 2009; Towa et al. 2020). EEIO models conceptualize waste not merely as a direct sectoral byproduct, but as a systemic consequence of production and consumption chains linked through complex economic interdependencies. Thus, even sectors perceived as low impact—such as services—may generate significant upstream waste through their demand for material- and energy-intensive inputs. Spain is one of the few EU countries that publish highly disaggregated physical waste data by service subsector (INE 2023a, 2023b, 2023c), making it a uniquely suitable case for testing the applicability of EEIO models to this underexamined but increasingly relevant sector.

The definition of waste adopted in this study refers exclusively to tangible solid waste, as defined by national and EU statistical frameworks. This includes municipal solid waste, packaging waste, construction and demolition waste, and industrial waste streams. A key conceptual distinction is drawn between direct waste—physically generated by a sector—and indirect waste, arising upstream from inputs associated with final demand. This distinction is essential for a comprehensive understanding of the service sector’s environmental footprint and is consistent with recent EEIO modeling advancements (Nakamura 2023). By treating waste as a residual output of sectoral production, the model reflects how economic productivity and material efficiency shape environmental outcomes. Sectors with higher input intensity or lower transformation efficiency tend to generate proportionally more waste, both directly and through supply chains. This structural logic underpins the rationale for using EEIO models to assess systemic inefficiencies.

To guide the analysis, this study tests two interrelated hypotheses. First, that the service sector, despite its relatively low direct waste generation, contributes substantially to overall waste through indirect channels, due to its dependence on upstream, resource-intensive supply chains. Second, that a significant portion of this indirect waste originates from foreign production, reflecting the globalized nature of service provision and trade. These hypotheses build on existing evidence from studies conducted in countries like Australia (Fry et al. 2016; He et al. 2020, 2018), France (Beylot et al. 2018, 2016), Poland (Gacek 2024), Spain (Ruiz-Peñalver et al. 2019), Taiwan (Chen et al. 2017; Liao et al. 2015), and global regions (Tisserant et al. 2017) and respond to the increasing need for more nuanced assessments of the service sector’s environmental footprint. This expectation is grounded in the structural composition of advanced economies, where service activities are increasingly interlinked with resource-intensive production sectors such as manufacturing, construction, and transport. Previous studies have documented these upstream linkages and their environmental consequences (Rosenblum et al. 2000), but few have quantified their waste implications with disaggregated data, particularly in national contexts like Spain.

## Materials and methods

### Data sources

Annex I of Regulation (EC) No 2150/2002 on waste statistics, as amended by Commission Regulation (EU) No 849/2010, mandates comprehensive data collection across all NACE Rev. 2 Sections (A to U). Until 2018, most EU countries estimated waste generation in the service sector using residual balancing methods. However, the progressive implementation of sector-specific surveys has substantially improved the granularity and reliability of waste statistics. Spain stands out as one of the few EU member states that publish highly disaggregated physical waste data by service subsector, thereby enabling a more accurate identification of indirect or upstream waste flows embedded in service activities.

This study relies exclusively on official, nationally harmonized secondary data sources. Specifically, we draw on three primary datasets published by the Spanish National Statistical Office (INE), which together provide a detailed breakdown of solid waste generation by economic activity:

- Data on waste generated by services and construction activities were obtained from the Survey on Waste Generation in Services and Construction, which covers NACE Rev. 2 Sections F to S (excluding O) (INE 2023a).
- Data on industrial waste were derived from the Survey on Waste Generation in Industry, covering Sections B to D of NACE Rev. 2 (INE 2023b).
- Waste generation in agriculture, forestry, and fishing (Section A) was drawn from the Environmental Accounts for Agriculture, Forestry, and Fishing, compiled by INE (INE 2023c).

All three sources report only tangible solid waste, as defined by Eurostat. Other environmental pressures—such as air emissions, wastewater, and energy losses—are excluded. The surveys provide activity-level data, enabling a robust assignment of waste flows to specific economic subsectors (INE 2023a, 2023b, 2023c). They are based on stratified sampling designs ensuring statistical representativeness at the national level. In the 2023 edition, the Survey on Waste Generation in Services and Construction covered more than 3500 establishments across NACE Rev. 2 sections F to S (excluding O), using stratified sampling based on firm size and activity. The overall sampling error for key waste indicators remained below 5%, according to the official methodological report. The data are also cross-validated with administrative registers (e.g., the Spanish Waste Management Registry) and benchmarked against structural business statistics, in line with Eurostat validation protocols under Regulation (EC) No 2150/2002.

Unlike earlier studies that relied on top-down allocations or imputation techniques, these datasets are compiled using exhaustive, survey-based methods, enhancing the reliability of the resulting waste accounts. The present study uses the 2023 edition of these surveys, which report waste generation for the year 2021, to ensure full temporal alignment with the FIGARO inter-country input–output tables employed in the EEIO model (Eurostat 2023). Maintaining this alignment is crucial for preserving consistency between environmental extensions and the underlying economic structure (Miller and Blair 2009). Nonetheless, we recognize the importance of updating future estimates as new data releases become available.

For the EEIO analysis, several global databases are available, each offering unique features and applications: the EORA database (Lenzen et al. 2013, 2012); the World Input–Output Database (WIOD), developed by Timmer et al. (2015); the Global Trade Analysis Program (GTAP) database, compiled by Aguiar et al. (2019); EXIOBASE, developed under the EXIOPOL project (Tukker et al. 2013); the Global Resource Input–Output Assessment (GLORIA), developed at the University of Sydney (Lenzen et al. 2021, 2017); and the OECD Inter-Country Input–Output (ICIO) database (OECD 2021). Each provides valuable insights into global trade and environmental interdependencies. The 2023 edition of the FIGARO inter-country input–output tables provides comprehensive inter-country supply, use, and input–output data for the EU27 member states, 18 major trading partners, and the rest of the world. The FIGARO tables are industry-by-industry, encompassing 21 industries, with transactions recorded in nominal million euros at basic prices (Eurostat 2023). Because FIGARO contributes directly to the OECD ICIO framework, it ensures methodological compatibility with global environmental accounting practices. This harmonization makes FIGARO especially suitable for tracing cross-border environmental flows, including indirect waste embedded in imported goods and services—an essential element of this study’s second hypothesis.

### Methodology

The input–output model (IO), originally proposed by Leontief in 1936 (Leontief 1936), provides a robust framework for analyzing intersectoral dependencies within an economy. In the 1970 s, Leontief extended this framework to incorporate environmental impacts, enabling assessments of resource use and waste generation across sectors (Leontief 1970). This theoretical foundation underpins the development of EEIO models, which have become standard tools for estimating indirect environmental pressures embedded in domestic and international supply chains (Nakamura 2023; Towa et al. 2020). Alternative approaches, such as physical flow accounting or life cycle assessment, offer valuable micro-level insights but lack the systemic traceability of upstream impacts. In contrast, EEIO models—grounded in Leontief’s intersectoral theory—are uniquely suited to capturing economy-wide indirect waste generation, particularly in service-based economies (Miller and Blair 2009; Nakamura 2023).

The fundamental IO equation relates total sectoral output (${x}^{r}$) to intermediate consumption (${A}^{r}$) and final demand (${y}^{r}$) for a given country (*r*):

1 $${x}^{r}={A}^{r}{x}^{r}+{y}^{r}$$

where ${x}^{r}$ is a vector of sectoral outputs in country *r*; ${A}^{r}$ is the matrix of intermediate consumption coefficients representing the inputs required to produce one unit of output; and ${y}^{r}$ is the vector of final demand in country *r.*

To capture both direct and indirect waste generation associated with domestic production and foreign trade, this study employs an environmental extension of the economic multi-regional input–output (MRIO) table. The environmentally extended MRIO table integrates monetary flows with sectoral waste generation data for Spain, aggregated across *n* countries and *k* industries. The structure of the extended MRIO table is shown in Table 1.

**Table 1:** Structure of the environmentally extended MRIO table

*Input*															
*Output*			Intermediate use							Final demand					*Output*
			Country 1			…	Country n			Country 1		…	Country n		
			Industry 1	…	Industry k	…	Industry 1	…	Industry k	H	…	…	H	…	
Intermediate inputs	Country 1	Industry 1	${z}_{11}^{11}$	…	${z}_{1k}^{11}$	…	${z}_{11}^{1n}$	…	${z}_{1k}^{1n}$	${y}_{1}^{11}$		…	${y}_{1}^{1n}$		${x}_{1}^{1}$
		…	…	…	…	…	…	…	…	…		…	…		…
		Industry k	${z}_{k1}^{11}$	…	${z}_{kk}^{11}$	…	${z}_{k1}^{1n}$	…	${z}_{kk}^{1n}$	${y}_{k}^{11}$		…	${y}_{k}^{1n}$		${x}_{k}^{1}$
	…	…	…	…	…	${z}_{ij}^{sr}$	…	…	…	…		${y}_{i}^{sr}$	…		${x}_{i}^{s}$
	Country n	Industry 1	${z}_{11}^{n1}$	…	${z}_{1k}^{n1}$	…	${z}_{11}^{nn}$	…	${z}_{1k}^{nn}$	${y}_{1}^{n1}$		…	${y}_{1}^{nn}$		${x}_{1}^{n}$
		…	…	…	…	…	…	…	…	…		…	…		…
		Industry k	${z}_{k1}^{n1}$	…	${z}_{kk}^{n1}$	…	${z}_{k1}^{nn}$	…	${z}_{kk}^{nn}$	${y}_{k}^{n1}$		…	${y}_{k}^{nn}$		${x}_{k}^{n}$
Value added			${v}_{1}^{1}$	…	${v}_{k}^{1}$	${v}_{j}^{s}$	${v}_{1}^{n}$	…	${v}_{k}^{n}$						
*Output*			${x}_{1}^{1}$	…	${x}_{k}^{1}$	${x}_{j}^{s}$	${x}_{1}^{n}$	…	${x}_{k}^{n}$						
Direct waste generation			${w}_{1}^{1}$	…	${w}_{k}^{1}$	${w}_{j}^{s}$	${w}_{1}^{n}$	…	${w}_{k}^{n}$						

Source: Own elaboration

The MRIO table includes the following elements:

- Intermediate consumptions ($${z}_{ij}^{sr})$$, which represent the intermediate consumption from industry *i* in country *s* to industry *j* in country *r*.
- Final demand (${y}_{i}^{sr}$), denoting the final goods or services produced by industry *i* in country *s* that are consumed to meet the final demand in country *r.*
- Value added ($${v}_{j}^{s})$$, reflecting the value added by industry *j* in country *s*.
- Total output $({x}_{j}^{s})$, accounting for all production by industry *j* in country *s*, including intermediate and final uses.

The intermediate consumption matrix ${A}^{sr}$ is derived by dividing each element of ${Z}^{sr}$ by the diagonalized output vector ${\widehat{x}}^{r}$ as follows:

2 $${A}^{sr}={Z}^{sr}{({\widehat{x}}^{r})}^{-1}$$

Each element ${a}_{ij}^{sr}$ within the matrix ${A}^{sr}$ quantifies the proportion of inputs from industry *i* in country *s* that are required to produce one unit of output in industry *j* in country *r*. This formulation provides a standardized view of input requirements across industries and countries.

Starting from Eq. (1), the standard IO model for country *s* can be expressed as

3 $${x}^{s}=\sum_{r=1}^{n}{A}^{sr}{x}^{r}+\sum_{r=1}^{n}{y}^{sr}$$

where ${x}^{s}$ denotes the vector of sectoral outputs in country *s*; ${A}^{sr}$ represents the matrix of intermediate consumptions, capturing the inputs from each industry in other countries necessary for producing outputs in country *s*; and ${y}^{sr}$ is the vector of final demand from country *s* to country *r*, reflecting the consumption of goods and services produced in country *s* by other countries.

Rearranging Eq. (3) yields

4 $${x}^{s}=\sum_{t=1}^{n}{B}^{st}{y}^{tr}$$

where $B\equiv {(I-A)}^{-1}$ represents the Leontief inverse matrix. The matrix ${B}^{st}$ quantifies the output required in producing country *s* for a one-unit increase in the final demand in destination country *r*.

To estimate the total (direct plus indirect) waste generated, we construct a direct waste generation vector $${(w}^{s}$$) analogous to that employed to the construction of the intermediate consumption matrix. The total waste generation ($${W}^{sr}$$) in country *s* resulting from demand in country *r* is calculated by multiplying the direct waste generation vector ($${w}^{s})$$ by Eq. (4):

5 $${W}^{sr}=\sum_{t=1}^{n}{({w}^{s})}^{\prime}{B}^{st}{y}^{tr}$$

This formulation allows the attribution of both immediate and upstream waste impacts to the final consumption of each sector, which is particularly important for service industries with low direct waste generation but high supply chain intensity, and to test our first hypothesis.

To analyze domestic and international contributions to waste generation, the Leontief inverse matrix ($$B)$$ and the final demand matrix $$(Y)$$ are decomposed into domestic and international components. The decomposition yield $${B}^{d}$$ and $${B}^{w}$$, capturing domestic international sectoral relationships, and $${Y}^{d}$$ and $${Y}^{w}$$, representing domestic and international final demand. Using these, the indirect waste generation can be expressed as

6 $${W}^{sr}={W}^{dd}+{W}^{dw}+{W}^{wd}+{W}^{ww}$$

where.

- ${W}^{dd}$: waste generated domestically due to domestic production, reflecting the internal waste generation from production activities within the country.
- ${W}^{dw}$: domestic waste generation due to foreign production demand, capturing how domestic inputs used to meet foreign demand leads to waste generation.
- ${W}^{wd}$: waste generated in foreign countries due to domestic demand, showing the impact of domestic consumption on foreign waste generation.
- ${W}^{ww}$: waste generated in foreign countries due to demand from other foreign countries, capturing impacts external to the domestic economy.

This decomposition is critical for testing the second hypothesis, as it reveals the transboundary nature of waste induced by the service sector, which is often masked in national statistics.

This study provides a cross-sectional snapshot based on 2021 data, the most recent harmonized and validated dataset available at the time of analysis. While annual fluctuations due to external shocks—such as the COVID-19 pandemic or energy price crises—may affect specific waste outputs, the EEIO framework is designed to capture structural patterns rather than short-term variability. These limitations are acknowledged and discussed in the concluding section. Additionally, the rapid digitalization of services presents emerging trends that could alter waste dynamics over time. Although these developments fall beyond the scope of this static model, they are identified as key priorities for future dynamic EEIO research.

Although the model does not incorporate site-level waste audits, it relies on official administrative statistics compiled from comprehensive surveys (INE 2023a, 2023b, 2023c) that follow Eurostat protocols and undergo external verification. Waste flows are uniquely assigned to sectors using NACE Rev. 2 classifications, avoiding overlaps. While part of the data is self-reported by firms, the surveys integrate company declarations with measured waste quantities and transfer documentation, and apply automated plausibility checks. These procedures are consistent with Eurostat’s quality control protocols. These protocols reduce potential sampling and non-sampling errors, including undercoverage of small service activities and misclassification across economic subsectors, thereby improving the accuracy of sectoral waste attribution. By integrating sector-specific physical waste data into a monetary IO framework, the model prevents double counting, in accordance with established EEIO modeling practices and Eurostat standards (Nakamura 2023).

## Results and discussion

The results are structured around a key analytical dimension of this study: the distinction between direct and indirect waste generation across economic sectors. This distinction is especially relevant for characterizing the environmental footprint of the service sector, which typically shows low direct material intensity but high upstream dependence on resource-intensive supply chains.

As shown in Table 2, waste generation in Spain is highly concentrated in a few sectors. Construction, waste and water management, and manufacturing together account for more than 84% of total waste. The construction sector alone is responsible for nearly half of all waste generated (47.34%), underscoring its systemic weight. This finding aligns with data reported by Eurostat (2024) and previous national studies in Australia and the UK (Fry et al. 2016; He et al. 2018; Salemdeeb et al. 2016). Notably, construction also displays the highest volume of indirect waste, reflecting strong intersectoral linkages with energy, materials, and heavy manufacturing.

**Table 2:** Total, direct, and indirect waste generated by sector, 2021

	Million tons			Percentage		
	Direct	Indirect	Total	Direct	Indirect	Total
Agriculture, forestry, and fishing	6.34	5.23	11.58	6.84	5.57	6.20
Mining and quarrying	2.95	0.24	3.19	3.18	0.25	1.71
Manufacturing	13.46	8.34	21.80	14.51	8.88	11.67
Energy	0.85	0.66	1.51	0.91	0.71	0.81
Waste/water	26.69	20.77	47.46	28.77	22.10	25.41
Construction	37.06	51.33	88.39	39.96	54.62	47.34
Services	5.41	7.40	12.81	5.83	7.88	6.86
Total	92.76	93.98	186.74	100	100	100

Source: Own elaboration based on official data from INE and Eurostat

In contrast, the service sector contributes a relatively modest 6.86% of total waste in absolute terms but displays a disproportionately high share of indirect waste—57.8% of its total. This confirms our first hypothesis: that the environmental burden of services is largely invisible in conventional statistics but emerges clearly when upstream flows are accounted for. This pattern is consistent with findings from Japan, where Nakamura (2020) shows that service industries, while low in direct emissions, are major contributors to incineration and landfill waste through upstream demand. Similar dynamics are observed in Belgium, where Towa et al. (2021) report that services such as retail, accommodation, and food are among the largest contributors to regional waste footprints, driven primarily by indirect flows.

Primary sectors such as agriculture, forestry, and mining display localized waste profiles, dominated by direct outputs. This reflects the nature of their activities, which are tied to raw material extraction and transformation with minimal upstream complexity. These sectoral profiles are consistent with those reported for Australia (Reynolds et al. 2016) and Taiwan (Liao et al. 2015).

Manufacturing, by contrast, presents a more balanced profile between direct and indirect waste generation, which reflects its dual role as both consumer and supplier of intermediate inputs. Waste and water management acts as a processing hub, handling residual flows from across the economy, and records the highest direct waste volume of any sector.

Disaggregating the service sector (Table 3) reveals marked internal differences. Wholesale and retail trade account for more than half of total service-sector waste, driven largely by indirect flows linked to packaging, logistics, and the upstream manufacturing of goods. This finding is supported by Fry and Schandl (2024), who highlight the growing contribution of service activities to plastic and packaging waste in Australia.

**Table 3:** Total, direct, and indirect waste generated by service industries, 2021

	Thousand tons			Percentage		
	Direct	Indirect	Total	Direct	Indirect	Total
Wholesale and retail trade and repair of motor vehicles and motorcycles	902.75	905.60	1808.36	16.70	12.23	14.12
Wholesale trade, except of motor vehicles and motorcycles	1310.08	2,147.94	3458.02	24.23	29.02	27.00
Retail trade, except of motor vehicles and motorcycles	1386.00	2075.94	3461.94	25.64	28.04	27.03
Transportation and storage	722.61	764.11	1486.72	13.37	10.32	11.61
Accommodation and food service activities	525.98	585.41	1111.39	9.73	7.91	8.68
Information and communication	44.47	52.40	96.87	0.82	0.71	0.76
Financial and insurance activities	21.17	18.93	40.10	0.39	0.26	0.31
Real estate activities, professional, scientific and technical activities, and administrative and support service activities	198.50	531.82	730.32	3.67	7.18	5.70
Education	53.84	57.20	111.04	1.00	0.77	0.87
Human health and social work activities	202.21	222.57	424.78	3.74	3.01	3.32
Arts, entertainment and recreation and other services activities	38.43	40.84	79.26	0.71	0.55	0.62
Services	5406.05	7402.75	12,808.79	100	100	100

Source: Own elaboration based on official data from INE and Eurostat

Transport and storage also emerge as major contributors (11.61% of service waste), largely due to indirect emissions associated with fuel use, vehicle production, and infrastructure demands. These results reinforce the importance of logistics efficiency and green mobility strategies, in line with the global waste footprint analysis by Tisserant et al. (2017).

Accommodation and food services, which represent key components of the tourism sector, account for 8.68% of total service-sector waste. Although less supply-chain intensive than trade or transport, they exhibit a substantial upstream footprint due to food provisioning, cleaning supplies, and infrastructure-related services. Much of this waste is generated indirectly through the production and distribution of food and beverages, packaging materials, and energy use in hospitality operations. In tourism-dependent regions, this subsector becomes particularly relevant for targeted waste prevention measures. Local sourcing, composting programs, and food waste reduction initiatives could significantly reduce the environmental footprint of tourism services, especially when coupled with circular procurement strategies and consumer awareness campaigns.

Knowledge-based services, including finance, ICT, education, and professional activities, contribute less than 5% of service-sector waste. These sectors, characterized by lower material intensity, support arguments for a transition toward less resource-dependent economic models (Meyer et al. 2020; Rosenblum et al. 2000). Their limited waste profile also indicates that digitalization and dematerialization offer promising avenues for decoupling economic activity from environmental pressure.

Table 4 presents a decomposition of indirect waste generation, categorizing it into four types: waste generated domestically due to domestic production (${W}^{dd}$); domestic waste driven by foreign production demand (${W}^{dw}$); waste generated abroad due to domestic demand (${W}^{wd}$); and waste generated abroad due to foreign demand (${W}^{ww}$). In most service subsectors, a majority of indirect waste is generated within Spain in response to domestic final demand (${W}^{dd}$). This supports our second hypothesis and aligns with findings from Belgium (Towa et al. 2021), where over 75% of service-sector waste is generated nationally. Retail, hospitality, and public services in particular exhibit highly localized waste footprints, suggesting that subnational and national waste policies can be effective in these areas.

**Table 4:** Decomposition of indirect waste generation by service industries, 2021

	Tons				Percentage			
	${W}^{dd}$	${W}^{dw}$	${W}^{wd}$	${W}^{ww}$	${W}^{dd}$	${W}^{dw}$	${W}^{wd}$	${W}^{ww}$
Wholesale and retail trade and repair of motor vehicles and motorcycles	698.10	112.75	81.58	13.18	77.09	12.45	9.01	1.45
Wholesale trade, except of motor vehicles and motorcycles	1017.11	874.82	137.63	118.38	47.35	40.73	6.41	5.51
Retail trade, except of motor vehicles and motorcycles	1317.23	659.03	66.44	33.24	63.45	31.75	3.20	1.60
Transportation and storage	409.18	222.23	86.00	46.71	53.55	29.08	11.25	6.11
Accommodation and food service activities	496.95	63.02	22.58	2.86	84.89	10.77	3.86	0.49
Information and communication	34.40	9.83	6.35	1.81	65.65	18.77	12.11	3.46
Financial and insurance activities	13.78	3.52	1.29	0.33	72.82	18.60	6.84	1.75
Real estate activities, Professional, scientific and technical activities, and administrative and support service activities	345.42	164.72	14.67	7.00	64.95	30.97	2.76	1.32
Education	54.73	2.22	0.24	0.01	95.68	3.88	0.42	0.02
Human health and social work activities	211.26	10.95	0.33	0.02	94.92	4.92	0.15	0.01
Arts, entertainment and recreation and other services activities	36.50	3.68	0.60	0.06	89.37	9.00	1.47	0.15
Services	4634.66	2126.77	417.72	223.59	62.61	28.73	5.64	3.02

Source: Own elaboration based on official data from INE and Eurostat

By contrast, wholesale trade and logistics display more internationally distributed waste profiles. In wholesale trade, over 40% of indirect waste is linked to foreign production or demand, indicating deeper integration in global value chains. Similar dynamics have been reported by Tsukui et al. (2022) for Japanese imports from China. These results suggest that circular economy strategies for globally integrated sectors must include cross-border cooperation, environmental standards in trade agreements, and extended producer responsibility across supply chains.

Public services—such as education, health, and cultural activities—exhibit overwhelmingly domestic waste profiles (${W}^{dd}$> 90%), which reinforces the potential for local governments to lead waste prevention initiatives through sustainable procurement, digitalization, and resource-efficient service provision (Jensen et al. 2013; Ruiz-Peñalver et al. 2019).

Taken together, the results provide robust support for both hypotheses. First, the service sector’s indirect waste footprint is substantial and underrecognized in standard waste accounting frameworks. Second, this footprint is largely domestic in origin, although certain subsectors—such as wholesale trade—show significant exposure to global drivers. These findings are consistent with previous EEIO-based studies in Japan, the UK, Spain, and Belgium (Nakamura 2020; Ruiz-Peñalver et al. 2019; Salemdeeb et al. 2016; Towa et al. 2021).

The observed heterogeneity across subsectors also highlights the importance of targeted, sector-specific waste strategies. High-volume, high-intensity services such as retail, logistics, and tourism-related activities like accommodation and food services call for upstream interventions (e.g., circular supply chains, packaging regulation, food waste reduction), while low-impact sectors like ICT and education offer opportunities for low-material service innovation.

## Conclusions and policy recommendations

This study provides a comprehensive evaluation of waste generation across industries in Spain, differentiating between direct and indirect (supply chain-related) waste and analyzing its domestic and foreign origins. Grounded in the principles of EEIO modeling and Leontief’s intersectoral theory, the analysis highlights how waste is generated and transmitted across economic production chains. The results provide empirical evidence that can inform the development and extension of Spain’s national sustainability strategies, particularly the España Circular 2030 agenda (MITECO 2020). While this framework outlines broad goals for reducing waste and promoting circularity, it still lacks specific targets tailored to the service sector. Our findings expose this gap and offer an empirical basis to address it, supporting Spain’s alignment with the EU Circular Economy Action Plan and the European Green Deal.

The findings confirm that the construction, manufacturing, and waste management sectors are primary contributors to total waste generation. Among these, the construction sector stands out as the largest source due to its material-intensive nature. In contrast, the service sector, while generating comparatively lower levels of direct waste, exhibits a considerable indirect impact driven by its complex supply chains. These results emphasize the need to conceptualize waste not merely as a direct byproduct of production, but as a systemic outcome of final consumption. Within services, wholesale, retail, and transport generate considerable indirect waste, while knowledge-based services (e.g., finance, communication) show markedly lower waste intensities. This heterogeneity confirms our first hypothesis and underscores the importance of designing differentiated, sector-specific waste prevention policies.

To address these differentiated waste profiles, Spain’s waste prevention efforts should prioritize upstream interventions in line with the EU waste hierarchy, focusing on avoiding waste generation at the source rather than relying solely on downstream management. In construction, this means promoting circular economy practices through stricter reduction targets, enhanced recycling obligations, and incentives for sustainable building materials and design. For the service sector, policies should prioritize the reduction of indirect waste through sustainable procurement practices, minimization of packaging waste, and the adoption of green logistics solutions. Economic incentives such as tax deductions for sustainable procurement, green public procurement criteria, and the extension of producer responsibility schemes to include packaging and e-waste from services could help align business incentives with circular economy goals. These measures should be supported by a clear governance framework in which governments provide regulatory guidance and enforcement, firms lead implementation and innovation, and civil society contributes through monitoring, awareness, and capacity-building. Additionally, voluntary corporate social responsibility initiatives, including environmental management systems and corporate waste reduction targets, can further support the transition to circular business models in services.

Addressing demand-side drivers is equally important. EEIO models link environmental impacts to final consumption, highlighting the need to incorporate sustainable consumption into waste strategies. In sectors such as retail, hospitality, and logistics, consumer behavior has a decisive influence on waste generation. Thus, awareness campaigns, food waste reduction programs, and over-packaging prevention can substantially complement supply-side interventions. Local initiatives, such as food waste prevention programs, community composting, and municipal recycling schemes, are particularly relevant for hospitality and retail subsectors, which exhibit high domestic waste generation.

Beyond domestic interventions, Spain has an opportunity to assume a leading role in promoting sustainable waste practices through international trade policy. As a high-income country with a significant share of upstream waste embedded in imports, Spain is well positioned to advocate for the integration of circular economy principles into trade agreements and multilateral frameworks. This includes advancing the harmonization of CE standards across EU and global markets, incorporating waste-related provisions into free trade agreements, and extending extended producer responsibility (EPR) schemes to imported goods. Recent studies emphasize the importance of aligning circularity goals with trade policy to avoid burden shifting and ensure that environmental and social objectives are met across supply chains (Barrie and Schröder 2022; Losa 2025). As Barrie and Schröder (2022) note, the success of a just transition to a circular economy depends on reconciling trade liberalization with environmental protection, particularly regarding secondary raw materials, end-of-life products, and transboundary waste flows. Spain’s engagement in EU Green Deal diplomacy and its leadership in national CE roadmaps can therefore contribute meaningfully to the development of more transparent, coherent, and equitable global waste governance.

Monitoring and accountability must also be strengthened. The European Environment Agency (EEA 2023) stresses the need for improved waste prevention indicators. Spain should develop standardized, sector-specific metrics and expand the integration of waste and economic data. Adopting best practices from other EU countries, such as sectoral benchmarks and dynamic monitoring tools, could significantly improve the policy cycle. Spain’s current circular economy strategy (MITECO 2020) lacks targeted measures for services; our findings support the creation of tailored regulatory frameworks, economic incentives, and voluntary agreements supported by robust data systems. Ensuring consistency with the EU Green Deal would enhance coherence across waste, climate, and resource efficiency agendas.

Evaluating the effectiveness of current policies is another priority. Our study highlights significant monitoring gaps, especially in the service sector. Improved tracking of public budgets for prevention, better survey instruments, and the inclusion of cost-effectiveness metrics would enable a more rigorous assessment of policy outcomes. This is particularly important given Spain’s lag in achieving municipal recycling targets and the current limitations of existing indicators. Improved data granularity is key. High-income economies like Spain account for over 30% of global waste (Kaza et al. 2018), much of it embedded in trade. Strengthening transparency in supply chains and extending EPR schemes to imported goods would allow Spain to promote circularity not only domestically but also internationally. These actions are consistent with the Spanish National Waste Plan (MITECO 2025) and emerging EU-level initiatives to monitor and prevent transboundary waste flows (EEA 2023).

This study has several limitations. First, the EEIO model relies on fixed technical coefficients and linear assumptions, limiting its ability to capture short-term changes in production structures or behaviors. As noted by Miller and Blair (2009), such models require expansion into dynamic or hybrid frameworks to enable sensitivity analyses. Second, the analysis focuses on gross waste flows without accounting for treatment pathways or recycling efficiencies. Third, while the Spanish case provides highly disaggregated data, its unique economic structure—especially the importance of tourism—may limit the direct applicability of results to other contexts. Fourth, the static model does not capture emerging trends such as digitalization or post-pandemic structural shifts, which could reduce service sector waste through dematerialization and remote service delivery. In addition, survey-based waste coefficients for emerging service activities (e.g., digital platforms, household-based care, gig economy services) may be affected by underreporting or classification challenges. These uncertainties can introduce biases in sectoral waste attribution, particularly where economic activity evolves faster than statistical coverage. While these limitations do not compromise the structural validity of the EEIO model, they do suggest caution when interpreting disaggregated results for rapidly evolving service subsectors. Fifth, the national-level focus limits regional insights, although future regionalized models could address this if subnational data become available. Sixth, the informal sector is not fully captured in official waste statistics, potentially leading to underestimation in small-scale service activities. Seventh, while this study provides a snapshot for 2021, it does not include a historical trend analysis, which could be addressed in future research using time-series data. Eighth, the study does not evaluate the effectiveness of existing sector-specific policies, which remains an open research need given identified monitoring gaps. Ninth, while the results are qualitatively consistent with findings from Australia, Japan, or the UK, cross-country quantitative validation is not performed due to differences in data availability and methodological conventions. Finally, the INE surveys report annualized data, which aggregate seasonal fluctuations. While this provides a representative yearly estimate, we acknowledge that future research could explore intra-annual variation using administrative microdata or time series for specific service subsectors.

Future research should build on this study by developing dynamic EEIO models, integrating life cycle inventory data, and incorporating material-specific waste flows such as plastics, food, and e-waste. Comparative analyses across EU economies using harmonized datasets and indicators are also needed to enhance international benchmarking. In line with Nakamura (2023), future work should aim to track waste at the product level to support extended producer responsibility schemes and cross-border circularity frameworks. Additionally, scenario-based modeling could explore the potential of digitalization and remote work to reduce waste in knowledge-based services. Equally important, future research should address the role of intangible environmental burdens—such as energy inefficiencies, digital pollution, and emissions from data infrastructures—which are not captured in current models but are increasingly relevant in service-oriented economies. Extending EEIO models or combining them with complementary methods to account for these non-material waste flows represents a key step toward a more comprehensive assessment of the environmental footprint of services.

## Data Availability

This study relies on publicly available datasets from the following sources: 1) Survey on waste generation in services and construction: covers waste generated by NACE Rev. 2 sections F to S (excluding O). Access provided by the Spanish National Statistics Institute (INE) at INE (2023a). 2) Survey on waste generation in industry: includes data for NACE Rev. 2 sections B to D. Available at INE (2023b). 3) Spanish environmental accounts: covers waste generation for NACE Rev. 2 section A (agriculture, forestry, and fishing). Accessible at INE (2023c). 3) FIGARO inter-country input–output tables (ICIO): provides inter-country input–output data for EU27 and key trading partners. Available at Eurostat (2023). These datasets are publicly available, and their use complies with the relevant terms and conditions of the original data providers. No proprietary or restricted-access data were used in this research. Further details on the data processing and analysis, as well as intermediate outputs, are available from the authors upon request.

## List of acronyms

EEIO: Environmentally extended input–output
EPR: Extended producer responsibility
EU: European Union
FIGARO: Full international and global accounts for research in input–output analysis
INE: Instituto Nacional de Estadística
IO: Input–output
MRIO: Multi-regional input–output
NACE: Nomenclature statistique des Activités économiques dans la Communauté Européenne
